# Benefits, barriers and enablers of maternity waiting homes utilization in Ethiopia: an integrative review of national implementation experience to date

**DOI:** 10.1186/s12884-022-04954-y

**Published:** 2022-09-02

**Authors:** Mekdes Kondale Gurara, Yves Jacquemyn, Gebresilasea Gendisha Ukke, Jean-Pierre Van Geertruyden, Veerle Draulans

**Affiliations:** 1 School of Public Health, College of Medicine and Health Sciences, Arba Minch University, Arba Minch, Ethiopia; 2 Global Health Institute, Faculty of Medicine & Health Sciences, University of Antwerp, Wilrijk, Belgium; 3 Department of Obstetrics and Gynecology, Universitair Ziekenhuis Antwerpen, Antwerp University Hospital, Edegem, Belgium; 4 Department of Midwifery, College of Medicine and Health Sciences, Arba Minch University, Arba Minch, Ethiopia; 5 Faculty of Social Sciences, Centre for Sociological Research, KU Leuven, Leuven, Belgium

**Keywords:** Maternal mortality, Maternal morbidity, Waiting home, Maternity waiting home, Access, Ethiopia

## Abstract

**Background:**

Though Ethiopia has expanded Maternity Waiting Homes (MWHs) to reduce maternal and perinatal mortality, the utilization rate is low. To maximize the use of MWH, policymakers must be aware of the barriers and benefits of using MWH. This review aimed to describe the evidence on the barriers and benefits to access and use of MWHs in Ethiopia.

**Methods:**

Data were sourced from PubMed, Google Scholars and Dimensions. Thirty-one studies were identified as the best evidence for inclusion in this review. We adopted an integrative review process based on the five-stage process proposed by Whittemore and Knafl.

**Results:**

The key themes identified were the benefits, barriers and enablers of MWH utilization with 10 sub-themes. The themes about benefits of MWHs were lower incidence rate of perinatal death and complications, the low incidence rate of maternal complications and death, and good access to maternal health care. The themes associated with barriers to staying at MWH were distance, transportation, financial costs (higher out-of-pocket payments), the physical aspects of MWHs, cultural constraints and lack of awareness regarding MWHs, women’s perceptions of the quality of care at MWHs, and poor provider interaction to women staying at MWH. Enablers to pregnant women to stay at MWHs were availability of MWHs which are attached with obstetric services with quality and compassionate care.

**Conclusion:**

This study synthesized research evidence on MWH implementation, aiming to identify benefits, barriers, and enablers for MWH implementation in Ethiopia. Despite the limited and variable evidence, the implementation of the MWH strategy is an appropriate strategy to improve access to skilled birth attendance in rural Ethiopia.

**Supplementary Information:**

The online version contains supplementary material available at 10.1186/s12884-022-04954-y.

## Background

Ethiopia, one of the countries in sub-Saharan Africa has been showing progress concerning a reduction in pregnancy-related maternal mortality that was 871 per 100,000 live births in 2000 to 412 per 100,000 live births in 2015 [1]. Even though it has been showing progress with its previous status, the country is still one of the 10 countries which had contributed to about 59% of the global maternal mortality in 2017 by losing the lives of 14,000 women as a result of pregnancy and childbirth-related complications [2]. Moreover, Ethiopia needs to accelerate its annual rate of maternal mortality reduction to achieve the SDG3.1 to record a maternal mortality rate of less than 140 per 100,000 live birth by 2030 [3].

As the majority of maternal deaths occur during childbirth and the immediate postpartum period, assistance during childbirth by skilled health personnel has been proved as one of the main strategies to decrease maternal mortality [4]. In Ethiopia however, the proportion of births assisted by skilled health personnel is less than half of the total births. Disparities in the utilization of skilled delivery care between urban and rural areas are wide and rural women, particularly those who are poor and illiterate, have limited access to healthcare with skilled health personnel [5].

Disparities in the use of this service have also been linked to supply-side limitations (access, quality, and affordability of the services) as well as demand-side limitations (mainly operating at the individual and community levels) according to prior studies in Ethiopia [6, 7]. Long distances from expectant mothers’ place of residence to the healthcare facilities, lack of transportation, and the mountainous terrain of most rural settings are among the barriers that contribute to the physical inaccessibility of skilled childbirth care services [5].

To address supply-side limitations, particularly physical accessibility, many efforts have been made by the government to avail primary health care units with basic emergency obstetric care for a maximum of 25,000 populations [8]. However, as the way of life of the rural communities in Ethiopia is not like that of towns, they are scattered over a wide geographic area and some with difficult mountains and valleys where it is not easy to construct roads, availing health facilities to all segments of the population [9]. Accordingly, the maternity waiting home strategy has been introduced to close the geographic gap between pregnant women and skilled childbirth care in a few Ethiopian hospitals in the 1980s [10, 11].

The strategy was implemented by setting up shelters near obstetric care facilities where pregnant women can stay during their final weeks of pregnancy and be quickly transferred to the care facility when they go into labor [10]. Maternity waiting home is a popular word used in the literature to refer to this accommodation facility, but similar facilities have also been referred to as maternity waiting homes or waiting areas [10, 12, 13]. As their names differ, so do the services they provide; some offer simple lodging, while others offer meal service, and still, others include health education and prenatal care [13, 14]. In terms of location, some are near or on the premises of health facilities where obstetric care is being offered, while others are standalone facilities offering accommodation services associated with the health facility with a referral link [12, 13, 15, 16].

Faith-based organizations have pioneered the construction of MWH in the late 1980s and later in 2015, it has been included in maternal health programs of the country as a means of overcoming distance-related barriers and increasing women’s access to life-saving emergency obstetric and neonatal care, particularly in the rural part of Ethiopia. Furthermore, in areas where the majority of births occur outside health institutions, all pregnant women are advised to stay at maternity waiting homes, especially those formerly regarded as “high risk” of developing childbirth complications [17, 18].

The National Reproductive Health Strategy 2016–2020 included a recommendation to scale up MWHs initiatives at health centers for women from remote areas to get quality of care on time, and adopted a target of 75% coverage by 2020. Accordingly, most of the health centers in Ethiopia have established MWHs [19], and in 2015, a standardized health facility guideline for the implementation was also approved by the Federal Ministry of Health. Some studies examined different aspects of MWHs in Ethiopia and underscored the variability in expansion, utilization, and benefits of maternity waiting homes across the country. Furthermore, previous research employed diverse methodological approaches thus a review that accommodates varied methodologies to identify the benefit, barriers and enablers to use the existing MWH services are needed. Thus, this integrative review will provide a comprehensive assessment of the literature on MWH strategy in Ethiopia.

## Methods

We adopted an integrative review to summarize literature to provide a more comprehensive understanding of MWHs implementation in Ethiopia. The study protocol was registered with the International Prospective Register of Systematic Reviews under the registration number CRD42019125308. Systematic reviews, while important to evidence-based practice, tend to focus on experimental studies, specifically randomized clinical trials, usually used to determine to evaluate the effectiveness of an intervention. However, the primary literature in the MWH aspect was diverse in methodology including descriptive, observational, and qualitative research. Therefore, an integrative literature review was chosen because it allows for a greater breadth of research to be analyzed and plays an important role in evidence-based practice in healthcare [20]. We adopted an integrative review process based on the five-stage process proposed by Whittemore and Knafl: Developing the review question, searching the literature, Data evaluation, Data analysis, and presentation of integrated findings [20].

### Databases and search

We conducted a systematic literature search across the three electronic databases: PubMed, Google Scholars and Dimensions, which encompass a wide range of research relevant to the healthcare domain.

Boolean connectors AND, OR and NOT were used to combine search terms and the keywords used were Health Services Accessibility“[MeSH Terms] OR “maternity waiting home*“[Text Word] OR “maternity waiting area*“[Text Word] OR “maternity waiting*“[Text Word]) AND (“Ethiopia“[MeSH Terms] OR “Ethiopia“[Text Word]). We have presented the detailed search strategies of PubMed in Additional file 1.

We also manually searched the reference lists of potentially relevant studies to find out studies that had not been identified during the search of electronic databases. We have contacted the corresponding authors for studies through the Research gate platform for the research we do have limited access due to a pay-wall restriction. We tried to employ a variety of search methods to ensure a broad representation of evidence from peer-reviewed journals and grey literature related to the subject matter.

### Eligibility criteria

The inclusion criteria for the type of document included (1) published in the English language, (2) from Ethiopia, (3) experimental, quasi-experimental or non-experimental design, (4) investigated maternity waiting homes benefit, barriers and enablers. Studies were excluded if they were reviews, protocols, commentaries, conference proceedings, and editorials.

### Data extraction and evaluation

**Fig. 1:**
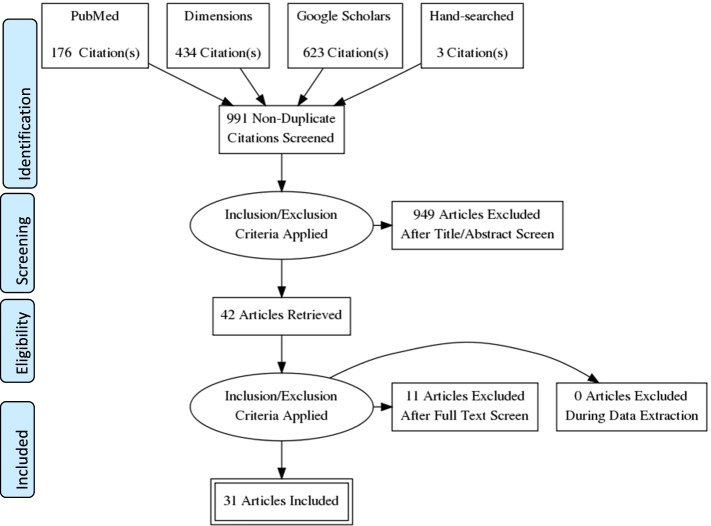
Article search and selection process using PRISMA flowchart

The database search generated 1234 records. Searches were imported into the Mendeley Desktop, an external citation manager, for further screening. After removing duplicates, 991 potential studies were identified and preliminary screening was done by checking the titles and abstracts of the remaining studies. Two authors independently screened the titles and abstracts against the inclusion criteria and identified 42 studies. In the case of disparities, a consensus was achieved by examining the full-text and collaborative discussion. After scanning reference lists of included and review papers, three studies were identified. Lastly, full-text reviews were conducted, and articles were removed if they did not meet the inclusion criteria. The final 31 articles were then, systematically reviewed; the screening and selection process is outlined in a PRISMA flow chart in Fig. 1. Information was pulled together in a summary matrix table (Tables 1 and 2) to highlight similarities and differences between studies. The extraction form included the following items: authors (publication year), the title of the study, purpose/aim, sample size and study population, research design and data collection, method of analysis, and key MWHs outcomes (barriers, benefits, and enablers).

**Table 1:** Summary of MWH studies included in the review in Ethiopia,2022

S.No	Author (year)	Purpose/Aim	Sample and study population	Study design and data collection	Methods of analysis	Location	Key MWHs related barriers and benefits of MWH use
1	Girma et al. (2021) [21]	To explore the experiences and challenges faced by women during the MWHs implementation initiative	12 pregnant women, 12 lactating women, 6 HEWs,6 midwives, 8 health center heads, 50 men	A multiple case study design with qualitative data collection methods	Thematic content analysis	Jimma Zone, Oromia	Lack of essential utilities; overcrowding; inadequate furnishing; and supplies and food
							Poor transportation services and the distance to MWH facilities
							Mothers have good perceptions of the services, yet the services are not satisfactory, and family reluctance is present.
							Lack of support to take care of children while pregnant mothers are staying in MWHs
							Poor interaction between healthcare workers and women in MWHS
							Lack of awareness about MWHs
2	Dalla Zuanna T, et al. (2021) [22]	To evaluate the effectiveness of MWH in reducing perinatal mortality in a secondary hospital	*N* = 3525 mothers 1175 cases and 2350 controls.	a retrospective study-nested case-control study and hospital / MWHs registry	multivariate logistic regression	Woliso, Oromoya	After adjusting for the confounder, the study highlighted a protective effect of MWH on perinatal mortality (OR = 0.70), and MWHs appear to reduce perinatal mortality by 55%.
							MWH users were older, came from rural areas, had a worse obstetric history, higher parity, and a higher number of previous cesarean sections than non-users.
							MWH users showed a significantly higher prevalence of all risk factors which are detectable during pregnancy
							MWH users show a similar or even lower prevalence of obstetric complications. However, among MWH users, the cesarean section delivery proportion was twice that of nonusers.
							The study also suggested the establishment of MWH, and there should be quality ANC in peripheral primary care clinics, where adequately trained healthcare professionals may recognize the occurrence of risk factors that may indicate admission to the MWH.
3	Gezimu1 et al. (2019) [23]	To assess the intention to use MWH and associated factors among pregnant women	*N* = 605 pregnant women	Community-based cross-sectional, face-to-face interview	Logistic regression	Kamba district, Southern Ethiopia	The study highlighted that 21.5% had stayed at MWHs, and 48.8% of pregnant women intended to use MWHs.
							Factors for the positive intention were occupation (being a government employee), previous childbirth history, the experience of MWH, direct and indirect subjective norms, and perceived behavioral control of the respondents.
							Those pregnant women who lived less than six months in the study area and those who delivered by cesarean section were excluded.
							Community disapproval, low self-efficacy, maternal employment, history of previous childbirth, and past experiences of MWHs utilization are predictors of intention to use MWHs.
4	Asmare et al. (2020) [24] (preprint)	To determine the proportion of and factors affecting male partners’ involvement in MWH utilization	*N* = 403	A community-based cross-sectional study	a multivariable logistic regression model	West Gojjam Zone, Northern Ethiopia	Male involvement in MWHs was found to be 55%.
							Young knowledge about MWHs, husband decision-making power, and receiving counseling during ANC about MWH were all predictors of male involvement.
							No standardized tool was used to measure the outcome variable
5	Endayehu et al. (2020 [25]	to assess pregnant women’s intentions to use MWHs and associated factors in East Bellesa district, northwest Ethiopia	*N* = 525 pregnant women	A community-based cross-sectional study, interviewer-administered structured	logistic regression	East Bellesa district, North Gondar	65.3% of pregnant women intended to use MWHs.
							Women’s knowledge, subjective norms related to women’s perceptions of social pressure, perceived behavioral control of women on the extent to which women feel confident to utilize, wealth status, decision-making power, attendance at antenatal care, and attitude towards women’s overall evaluation of MWHs were significantly associated with the use of MWHs.
							Efforts shall be made to improve women’s awareness by providing continuous health education during antenatal care visits, devising strategies to improve women’s wealth status, and strengthening decision-making power may enhance their intention to use MWHs.
6	Obola et al. (2020) (preprint) [26]	To assess intention to use MWH and associated factors among pregnant women	*N* = 556	A community-based cross-sectional study, face-to-face interview	multivariable logistic regression	Hadiya Zone	44.6% of pregnant women intended to use MWH.
							Mother attendance of formal education, 3 + received ANC care, MWH stays, and favorable and perceived behavior control were factors of the intention to stay at MWH.
7	Nigussie et al. (2020) [27]	To assess the predictors of intention to use MWH	*N* = 829 women	A community-based cross-sectional study	hierarchical linear regression	Bench Maji Zone, Southwest Ethiopia	42.6% of the study participants had an intention to use MWH, and 39% of the respondents ever used MWH
							ANC utilization, attitude toward MWH, subjective norm, and perceived behavioral control were significant predictors of intention to use MWH.
8	Hailu et al. (2021) [28]	To investigate the use of health institution for delivery and factor that determines institutional delivery.	*N* = 426 142 cases and 284 controls	A community-based unmatched case-control study	backward stepwise logistic regression	Omo Nada district, Ethiopia	61.4% of the women who used MWHs have delivered in health facilities
							Husbands and mothers themselves have a negative attitude towards MWHs. Therefore, the significant determinants for institutional delivery were mothers’ education, husband education, and ANC follow-up besides staying at MWH.
							The use of MWHs increased institutional delivery service use.
							However, it remains unclear whether the two groups are comparable or not other than the presence of disease in cases (health facility delivery) or the absence of disease in controls (home delivery).
9	Getachew et al. (2021) [29]	To describe establishment cost, essential services provided and operating costs of MWHs (MWH)	*N* = 812 postpartum women 8 eight health facilities with MWHs	cross-sectional study, MWH users exit interviews, observation, record review	descriptive analysis	Gurage Zone of Ethiopia	Most MWHs provide essential clinical services and basic amenities. However, not all MWHs provide meals to the users.
							The majority of the cost of MWH was attributed to building construction costs. However, if the building cost is annualized, the unit cost of an MWH service is in an acceptable range which encourages the government to consider expansion of the service in rural areas.
							The type of MWH varied between the sites, from traditional huts to semi-modern or modern and well furnished, built from natural materials or concrete bricks.
							The average initial costs for construction and furnishing an MWH were $ 2,245 US. The annual operating cost of an MWH was $2,882 US.
10	Erickson et al. (2021) [15]	To assess factors influencing MWH use, as well as the association between MWH stay and obstetric outcomes in a hospital in rural Ethiopia	*N* = 489 women gave birth at the hospital, 93 MWH users	a mixed methods observational cohort study/ Medical records, Key informant interviews of a convenience sample	Logistic regression, a thematic analysis performed	Gurage in southwest Ethiopia	Of four hundred eighty-nine births, only 19.0% of MWH stay. Clients were admitted to MWH for both medical and socio-cultural-economic reasons.
							Opportunity costs as the result of staying at MWH due to missed work and need to arrange for care of children at home, long travel times, and lack of entertainment during stay
							MWH users were significantly more likely than non-users to have a cesarean section.
							MWH use was associated with a 77% lower risk of childbirth complications, a 94% lower risk of fetal and newborn complications, and a 73% lower risk of maternal complications compared to MWH non-users
							Users were less likely to experience obstructed labor or stillbirth; no cases of uterine rupture among users, whereas nine women experienced it among non-users (2.3% of 396) though the difference was not statistically significant.
							Birth weight and 5-min Apgar scores were also higher in offspring of MWH users.
							If communities have involved in designing MWHs, they may serve as centers for women’s empowerment, education, and income generation, impacting women and families far beyond birth outcomes.
11	Asnake et al. (2020) (preprint) [30]	To assess the contribution of MWHs in immediate Postpartum FP in Ethiopia	*N* = 884 postpartum women	a comparative cross-sectional study design, interview	logistic regression	USAID Transform: Primary Health Care project in 4 regions	41% of women had used MWHs before delivery. Of the women who used MWHs and received postpartum family planning counseling, more than half (54%) accepted family planning.
							The prevalence of IPPFP use among women who used MWHs was 44%, and 36% among those who did not use MWHs.
							It is agreed that providing a comprehensive package of services, including FP counseling and services in MWHs, would positively impact FP uptake.
							It is challenging to attribute the change in the percentage between users and non-users due to MWH.
12	Gurara et al. (2021) [31]	To assessed barriers to MWHs	*N* = 807	Mixed methods design, both quantitative and qualitative methods of data collection employed.	logistic regression analysis	Gamo Zone	8.43% of the participants used MWHs during their last pregnancy.
							Out of the 68 women, 67% mentioned transportation problems, 75% absence of food catering at the MWHs, and 16% poor availability of utensils and attitudes of the providers toward the expectant mothers were the main challenges they faced.
							Previous childbirth complications, poor transport alternatives, long-distance travels through mountainous terrains to the facilities, and their husbands’ consent as factors
							The women’s economic status, decisions made jointly with male partners (husbands) for an obstetric emergency, history of previous institutional childbirth, BPCR practice, history of previous childbirth complications, < 2 h travel distance to the nearest HI, and ease of access to transport in case of obstetric emergency
13	Teshome et al. (2020) (preprint) [32]	To assess MWH utilization and associated factors among women	*N* = 530 women	Community-based cross-sectional study, face-to-face interview	logistics regressions	Arsi Zone, Oromia,	23.6% of the respondents used MWH
							Traveling time < = 60 min from a nearby health facility, women’s decision power, no antenatal care, and having more than three children were factors in using MWH
							The absence of someone who cares for children at home (31.5%), past favorable conditions during home delivery (26.2%), and no means of transport (20%) was reported as major challenges for not using MWH.
14	Kurji1 et al. (2020) [33]	To evaluate the effectiveness of functional MWHs combined with community mobilization by trained local leaders in improving institutional births	24 PHCUs and 7593 women were	A parallel, three-arm, stratified, cluster-randomized controlled trial design,	intention to treat approach	Jimma zone Ethiopia	The combined MWH & leader training and the leader training alone intervention led to a small but non-significant increase in institutional births compared to usual care.
							In the end line, institutional births were slightly higher in the MWH + training (54%) and training only arms (65%) compared to usual care (51%).
							MWH use at baseline was 6.7% and 5.8% at the end line. Both intervention groups exhibited a non-statistically significantly higher odd of institutional births than usual care.
							Low MWH use has often been linked to the poor quality of services offered (15% of women in the end line from the MWH + training arm were dissatisfied with the quality of services.
							Travel time and distance have been reported to be inversely correlated with MWH us
							Implementation challenges and short intervention duration may have hindered intervention effectiveness.
15	Tenaw et al. (2020) (preprint) [34]	To estimate the magnitude of MWH utilization and identify its associated factors in Sidama Zone	*N* = 748	Community-based cross-sectional study, Interviewer administered	Multivariable logistic regression analysis	Sidama zone	The utilization of MWH was 67.25%. Young age, socioeconomic status (high monthly income), and good knowledge make them more likely to use MWH.
							Women who knew MWH, women who had a husband who could read and write, and women who were protestant religion followers have higher probabilities of MWH utilization.
							Health education about MWH utilization, spouse education, and women’s economic empowerment are crucial to enhancing MWH utilization.
16	Kebede et al. (2020) [35]	To explore the factors influencing women’s access to the MWHs in rural Southwest Ethiopia.	*N* = 30 4 FGDs and 18 IDIs	A community-based cross-sectional study, qualitative data	thematical analysis	Southwest Ethiopia	Women were interested in MWHs and aware of their existence in their immediate vicinity. However, women did not understand the aims and benefits of MWHs.
							Health information dissemination and referral linkages by frontline health workers enabled women to timely access the MWHs.
							At the facility level, there were attempts to improve the acceptability of MWHs by allowing women to choose their delivery positions. However, participants claimed a lack of privacy and the presence of disrespectful care
							Physical barriers (long-distance, unavailability of transport options & unfavorable roads) were considered potential problems for women residing in remote areas.
							MWH users mentioned absences of sufficient basic facilities, poor quality and varieties of food. Because of insufficient facilities, the cost of living was high for most users. Therefore, the communities try to overcome the indirect costs through in-kind contributions and cash.
17	Selbana et al. (2020) [36]	to assess the utilization of MWHs and associated factors.	*N* = 379 women	A community-based cross-sectional study, face-to-face interview	Logistic regression analysis	Keffa Zone	42.5% of pregnant women stayed at MWHs.
							Women’s decision-making capacity, women having someone who can care for their children and husband at home; MWHs offering food service; offering and allowing women to practice their cultural ceremony (allowing to cook their food type, porridge, coffee, Etc.) and women’s attitude towards MWHs were factors significantly associated with the utilization of MWHs.
							Integrating culturally sensitive and supportive maternity services and a participatory community approach would increase the utilization of MWHs and consequently contribute to achieving the SDGs related to maternal health.
18	Getachew et al. (2020) [37]	To identify the influence of perceived geographic barriers to the utilization of MWHs	*N* = 716 women (358 were MWH users)	Observational cross-sectional study	Directed Acyclic Graph concept and multiple logistic regression	Gurage Zone of Ethiopia	MWH users had lower odds of having delivery complications.
							Women with pregnancy complications who did not use MWH were more likely to develop delivery complications. In addition, women with delivery complications had higher odds of undergoing cesarean delivery and neonatal death.
							Women who gave birth in non-cesarean section facilities had lower odds of delivery complications.
							This study strengthens the evidence of MWH utilization as a helpful strategy to overcome geographic barriers and lower delivery complications.
							Geographic barriers influenced the utilization of MWH. The women who used MWH had lower delivery complications.
19	Vermeiden et al. (2019) (preprint) [38]	To explore perspectives on MWH (MWH) utilization and facility births from the perspectives of community members and healthcare workers	*N* = 74 33 in-depth interviews and five focus group discussions	A qualitative study	Framework analysis	Gurage zone	Facility births were considered more common, yet uncomplicated births preferably took place at home. Ambulance services were highly appreciated in case of complications, while MWHs were unknown to most community members, and husbands were likely to object to their wives staying at MWHs.
							Many community members reported negative experiences at health facilities, especially hospitals. In contrast, MWH users recounted a positive experience and recommended it to others.
							Community networks have facilitated MWH stays and facility births through saving schemes and household support.
							HCWs were also optimistic about the quality of care, but examination areas needed improvement. In addition, being overworked, underpaid, and undertrained undermined the quality of care.
							Providing high-quality, compassionate care at health facilities was crucial to MWH use and facility births. In addition, community networks and health education may potentially overcome existing barriers to MWH use and facility births.
20	Kurji et al. (2020) [39]	to identify individual-, household- and community-level factors associated with MWH use in Ethiopia	*N* = 3784 women	Cross-sectional analysis of baseline household survey data	multi- variable generalized linear mixed-effects regression	Jimma zone	7% of women reported past MWH use. Housewives, women with companions for facility visits, wealthier households, and those with no health facility nearby or living > 30 min from a health facility had significantly higher odds of MWH use.
							Education, decision-making autonomy, and community-level institutional births were not significantly associated with MWH use.
							The short duration of stay and failure to consider MWH as part of birth preparedness planning suggests that local referral and promotion practices need investigation to ensure that women who would benefit the most are linked to MWH services.
21	Getachew et al. (2019) [40]	To compare the health care expenditures between MWH (MWH) users and nonusers in Ethiopia	*N* = 812 postpartum women	Cross-sectional study, face-to-face interviews	quantile regression to explore associated factors	Gurage Zone of Ethiopia	There were significantly higher out-of-pocket payments (OOP), women’s costs, total costs, and overall costs among MWH users compared with nonusers, regardless of the duration of their MWH stay.
							The MWH users were more likely to have higher OOP payments than MWH nonusers in linear and quantile regressions for both unadjusted and adjusted analyses.
							Higher OOP payments were observed for longer distance traveled and cesarean section (CS) delivery women. In addition, using public transportation was significantly associated with higher OOP payment in all quantile levels.
							The utilization of MWH was associated with higher OOP payments. Higher OOP payments for delivery care among MWH users were observed in all quantile of expenditure.
22	Kebede et al. (2019) [41]	to assess women’s MWH satisfaction	*N* = 362 women	Cross-sectioal study, face-to-face interviews	Multiple linear regressions	Jimma Zone	A 68.8% level of MWH satisfaction was reported. Higher mothers’ satisfaction was from social support aspects: one to five women’s network (89.5%), cleaner/servant in MWH (88.9%), and husband (87.3%).
							Lower satisfaction was from the ambulance (24%), recreational (38.5%), and food (49.4%) services and utensils in MWH (56.2%). Nearly 2/5th of users claim they do not come again and recommend MWH to others.
							Women’s overall satisfaction with MWH was predicted by length of stay in MWH (≤ 14 days), utensils in MWH, services (prenatal, food, sanitation, recreational), social supports (family, women’s 1–5 networks, and servants) and interpersonal communication with HCWs.
23	Bergen et al. (2019) [16]	To explore the barriers and enablers that Health Extension Workers (HEWs) encounter when engaging with communities about MWHs.	36 HEW	Across sectional study, Qualitative, in-depth interview	thematic content analysis	Jimma zone	HEWs reported various factors that determined MWA use, including the number of children at home, previous childbirth experiences, community support networks, family decision-making practices, the availability and acceptability of health services, geographical access, and health beliefs.
							HEWs worked to increase the use of MWAs by engaging with husbands and communities, raising awareness in target groups of women, and managing community participation.
							At the individual level, HEWs reported that some women did not see the importance of using an MWA, while others were compelled to prioritize remaining at home to care for their families. Within families and communities, male partners and support networks appear instrumental in enabling or deterring MWA use. Prominent factors associated with intermediate and structural determinants of health included functionality and acceptability of the MWA and adjacent health facility, geographical access, and cultural/social norms
							Though MWHs, by design, aim to address geographical barriers to facility birth, access to MWH itself frequently emerges as a barrier. For example, while ambulances, if available and functioning, may be a viable transportation option for women during labor, this service is not available for women to attend MWHs.
							Compared with decisions about place of birth, decisions about MWA use entail additional considerations, as women spend a greater amount of time away from home and adapt to different living conditions at an MWA.
24	Vermeiden et al. (2018) [42]	To describe facilitators for MWH utilization from the perspectives of MWH users and health staff	FGD = 28 participants IDI = 7 participants *N* = 244 respondents MWH users	mixed-methods design, review of the record, FGD, in-depth interview, and observation	content analysis, descriptive statistics, and data triangulation	Gurage zone	Perceived high quality of care at the health facility, awareness of their high-risk status, support in overcoming barriers (supportive husbands), and women’s groups were facilitators of MWH utilization. In addition, community and facility-level integrated health services were also facilitators for using MWH.
							Barriers to utilization existed (no cooking utensils at the MWH; attendant being away from work), but users considered these necessary to overcome for the perceived benefit: a healthy mother and baby.
							If providing high-quality EmONC and integrating health services are prioritized, MWHs have the potential to become an accepted intervention in (rural) communities. Only then can MWHs improve access to EmONC
							It was suggested that health education is crucial to facilitate MWH utilization, including clear communication to women and their families about the indications for an MWH stay.
25	Vermeiden et al. (2018) [43]	To describe factors and perceived barriers associated with the potential utilization of an MWH	428 recently delivered and pregnant women	A community-based cross-sectional study, interview	Logistic regression	Gurage zone, southern Ethiopia	7.0% had heard of MWH. In addition, 55.1% of the women showed a positive intention to stay at MWHs after explaining the concept to them.
							Last childbirth complications and envisioning fewer barriers to staying at MWH were associated with the positive intention to stay at MWH.
							Unless community awareness of preventive maternity care increases and barriers for women to stay at MWHs are overcome, these facilities will continue to be underutilized, especially among marginalized women.
26	Gebremeskel et al. (2018) [44] (Thesis)	To assess the effect of MWH utilization on maternal and perinatal health outcomes	MWH user (330) and non-user (343)	Retrospective Cohort Study	Life table and Cox proportional hazard regression	Tigray region	The incidence rates of maternal complications, perinatal death, and complications were significantly lower among the MWH users than non-users.
							Users and mothers who gave birth to twins were the independent predictors of the maternal complication.
							Newborns born from the user, born from mother who had experienced an obstetric complication, rural residents and newborns weighted < 2500 gm were the independent predictors of perinatal death and complications.
27	Braat et al. (2018) [14]	To examine the impact of a MWH by comparing pregnancy outcomes between users and non-users at hospitals with and without an MWH	*N* = 550 (244 MWH users and 306 non-MWH)	A retrospective cohort study	χ2 and OR	Gurage zone, southern Ethiopia	MWH users were less educated, poorer, and had to travel longer to reach a hospital compared with non-users
							While poverty and inequity are factors known to negatively impact the survival of women and neonates, the more vulnerable group of women had better birth outcomes than women with higher socioeconomic status who did not use an MWH.
							Between 2011 and 2014, all maternal deaths and nearly all stillbirths and uterine ruptures occurred among women who did not use the MWH
							High-risk pregnant women that used an MWH in rural Ethiopia had less favorable sociodemographic characteristics but better birth outcomes than women who gave birth at the same hospital but did not use the MWH and women who gave birth at a hospital without an MWH.
							The use of an MWH appears to improve birth outcomes.
28	Meshesha et al. (2017) [45]	The Role of MWH in improving Obstetric Outcomes	*N* = 516 mothers	Hospital-based record review	χ2, independent samples t-test	Jinka Zonal	16.7% had stayed at MWH, and the rest were directly admitted to the hospital (516 mothers).
							Most mothers who came to the labor ward via the MWA were pregnant women living in rural areas.
							The prevalence of bad obstetric outcomes was 61.2% among direct admission and 33.7% after they stay at MWH.
							Descriptive results showed that limited women’s ability to access facility-based obstetric care and attributing the percentage change to the utilization of MWH is unscientific.
29	Gaym et al. (2012) [46]	To describe the current status of MWH services in Ethiopia.	9 MWH 74 mothers	Cross-sectional: site visits and documentation review using a checklist	Thematic analysis	Nationwide	Seven MWHs required the clients to cater for their food, firewood, and clothing supplies, providing only kitchen space and a few kitchen utensils.
							The client admitted to the MWH was as far from 400 Km away to obtain services, and major indications for admission were previous cesarean Sect. 34%; previous fistula repair 12%; multiple pregnancies, 12% and mal-presentations 8%.
							Lacks standardization and institutionalization across all the facilities
							Selection bias, sample size adequacy, and group comparability are all uncertain.
30	Kelly et al. (2010) [13]	To describe maternal mortality and stillbirth rates among MWH users and non-MWH over 22 years.	*N* = 24 148 deliveries (6805 admitted via MWA and 17 343 admitted directly)	Retrospective cohort study, Data abstracted from routine hospital records.	Descriptive	Gurage zone	Maternal mortality and stillbirth rates were substantially lower in women admitted via MWA. At least part of this difference is likely accounted for by the timely and appropriate obstetric management of women using this MWH facility.
							The need for coordination between the community and appropriate secondary care facilities in operating an effective MWA was recommended.
							A descriptive comparison of perinatal outcomes and uncertainty around the comparability of groups might be the limitation of the study.
31	Poovan et al. (1990) [47]	To examine the impact of MWH on maternal health	No information	retrospective, record review	Descriptive	Gurage zone	A retrospective hospital-based study of pregnancy outcomes among MWH users versus those who went directly to the hospital
							There were no maternal deaths among the 142 MWH users, but there were 13 maternal deaths among the 635 MWH nonusers, with no statistically significant difference in the proportion of operative deliveries between the two groups

**Table 2:** Concept matrix mapping on benefits and barriers to stay at MWHs in Ethiopia from the included studies, 2022

S.No	Author (year)	Benefits			Barriers											
		**Low maternal and neonatal death and complication**	**Better access to health services**	**Better access to health information**	**Lack of basic utilities**	**Overcrowding**	**Meal services**	**Poor transportation services**	**Distance to MWH and unfavorable roads.**	**Lack of support**	**Poor provider-client interaction**	**Lack of awareness and poor attitude**	**Socidemo-economic**	**Previous experiences**	**Perceived quality of care**	**Higher Out of Pocket payment**
1	Girma et al. (2021) [21]				•	•	•			•	•	•				
2	Dalla Zuanna et al. (2021) [22]	•											•	•		
3	Gezimu1 et al. (2019) [23]											•	•	•		
4	Asmare et al. (2020) [24]									•		•	•	•		
5	Endayehu et al. (2020) [25]											•	•	•		
6	Obola et al. (2020) [26]												•	•		
7	Nigussie et al. (2020)[27]											•				
8	Hailu et al. (2021) [28]		•									•				
9	Getachew et al. (2021) [29]					•										
10	Erickson et al. (2021) [15]	•							•	•						•
11	Asnake et al. (2020) [30]		•								•					
12	Gurara et al. (2021) [31]				•		•	**•**	•	•	•		•	•		
13	Teshome et al. (2020)[32]		•							•		•	•	•		
14	Kurji1 et al. (2020) [33]		•	•				**•**	•						•	
15	Tenaw et al. (2020) [34]											•	•			
16	Kebede et al. (2020) [35]				•		•	**•**	•			•			•	•
17	Selbana et al. (2020) [36]						•			•		•	•		•	
18	Getachew et al. (2020) [37]	•	•						•							
19	Vermeiden et al. (2019)[38]			•												
20	Kurji et al. (2020) [39]												•			
21	Getachew et al. (2019) [40]							•	•							•
22	Kebede et al. (2019) [41]											•			•	
23	Bergen et al. (2019) [16]			•				•	•			•	•	•		
24	Vermeiden et al. (2018) [42]														•	
25	Vermeiden et al. (2018) [43]		•	•			•			•						
26	Gebremeskel et al. (2018) [44]	•														
27	Braat et al. (2018) [14]	•	•													
28	Meshesha et al. (2017) [45]		•													
29	Gaym et al. (2012) [46]		•													
30	Kelly et al. (2010) [13]	•														
31	Poovan et al. (1990) [47]	•														

### Study quality and bias

While it is agreed that potential studies for inclusion in the review should be evaluated for quality and bias, the best approach for assessing research quality in an integrated review is still up for debate. To evaluate the various forms of the methodology employed in the studies, various sorts of quality criterion tools can be applied. To assess the quality of the research included, Joanna Briggs Institute Critical Appraisal tools for qualitative and quantitative study [48]. Studies with statistically insignificant or negative outcomes, or study topics that may not be relevant to the journals’ scope, are less likely to be published than studies with significant or positive results. As a result, many completed studies are never published. To make the evaluation more comprehensive, we have also included unpublished studies (dissertations, theses, conference papers, and preprints after they were evaluated against the inclusion criteria [48].

### Data analysis and synthesis of results

Data analysis in research reviews requires that the data from primary sources are ordered, coded, categorized, and summarized into a unified and integrated conclusion about the research problem [20]. A constant comparison method: extracted data are compared item by item so that similar data are categorized and grouped. The method consists of data reduction (qualitative, quantitative, and mixed), data display, data comparison, conclusion drawing, and verification were also made [20]. Given the diversity of quantitative studies in terms of research questions, methods, samples, study settings, outcomes and outcome measures used, we undertook a narrative synthesis. The findings from the narrative synthesis of quantitative findings and the thematic analysis of the qualitative findings were then synthesized to identify common themes. A summary table was generated synthesizing the data from included studies (Table 1).

## Results

### Study characteristics

Analysis of the study characteristics presented in Table 1 revealed that 18 of the 31 articles reviewed were from the southern part of the country, 8 from Oromiya, 3 from the northern part, and the remaining two articles based on two or more regions. Though our search was not restricted by publication dates, the search produced studies published from 1990 to 2021. Thirty of the thirty-one studies were published between 2010 and 2021, which clearly shows an increasing demand and trend in the examination of MWHs. Of the 31 studies that met the inclusion criteria, 26 studies utilized a variety of quantitative study types including mixed-method designs.

Of the 31 studies that met the inclusion criteria most focused on the intention to use MWHs and factors that affect MWH use [23, 26, 28, 30–32, 35, 39, 43]. Few studies have looked retrospectively at the impact of MWH on maternal health and service utilization [13–15, 22, 47]. Only one clustered controlled trial looked at how upgrading MWH affects institutional delivery utilization, and the results were promising, though statistically insignificant [31, 33]. The use of MHW was found to be low in all studies.

### Key themes

The key themes reflect the review aims were the benefits, barriers and enablers of MWH utilization. Thematic analysis revealed 10 major sub-themes (Table 2). The themes of benefits were lower incidence rate of perinatal deaths and complications, the low incidence rate of maternal complications and deaths and improved access to maternal health care. The themes associated with barriers to staying at MWH were distance, transportation, financial costs (higher out-of-pocket payments), the physical aspects of MWHs, cultural constraints and lack of awareness regarding MWHs, women’s perceptions of the quality of care at MWHs, and poor provider interaction to women staying at MWH. Enablers to pregnant women to stay at MWHs were availability of MWHs which are attached with obstetric services with quality and compassionate care and additional promotional intervention at MWHs.

### Benefits of MWHs

#### Lower incidence rate of maternal death and complications

Seven of the studies showed that pregnant women admitted to obstetric hospitals through MWH had a lower risk of maternal complications and death [13–15, 37, 44, 45, 47]. MWH utilization was linked to a 77% lower risk of childbirth complications [15], users were less likely to have obstructed labor, and there were no cases of uterine rupture among MWH users, compared to nine cases among non-users [15, 37]. According to Braat et al. study, all maternal deaths and uterine ruptures occurred during the study period among women who did not utilize the MWH [14]. Moreover, Poovan et al. had reported that there were no maternal deaths among the 142 MWH users, but there were 13 maternal deaths among the 635 MWH nonusers [47].

#### Lower incidence rate of perinatal death and complications

MWHs were found to reduce the rate of perinatal problems and death among pregnant women admitted directly to hospitals and those who stayed in MWHs [13–15, 22, 44, 45, 47]. According to Zunna et al., MWHs have a protective effect against prenatal death, and it appears to reduce perinatal mortality by 55% [22]. Moreover, MWH users had normal birth weight and Apgar scores newborn, and they were also less likely to have a stillbirth [15]. Perinatal death and complications were much lower in MWH users than in non-user [44], stillbirth rates were significantly lower in women admitted via MWH [13].

#### Better access to maternal health services and health information

Nine of the studies showed a correlation between MWHs and improved access to maternal health services, particularly skilled childbirth care [14, 28, 30, 32, 33, 37, 43, 45, 46]. MWHs strategy has been a means for increasing the demand for maternity care, improving geographic access to childbirth, addressing the second delay, delay in reaching a health center and enabling more timely and comprehensive obstetric care [14, 28, 33, 37, 39, 46]. Four of the included studies reported that women who stay at MWH have better access to health information than women who did not stay at MWH [16, 33, 43, 49]. Those mothers staying at MWHs can share experiences with other pregnant mothers and receive postpartum health education about family planning, infant feeding, and the importance of maternal health services utilizations including PNC services for both mothers and new-borns [16, 33, 43, 49].

### Barriers to using MWHs

#### Distance

Factors that influenced a woman’s decision and ability to stay at MWH during her pregnancy included distance (accessibility) and transport (absence of ambulance services to and from MWHs) [15, 16, 31, 35, 37, 39, 42]. Women who had long travel times to reach health facilities were more often advised to stay in MWH to overcome a geographic barrier, but geographic barriers also had an impact on MWH use [37]. Women who had long travel tomes to reach health facilities were more often advised to stay in MWH to overcome a geographic barrier, but geographic barriers also impacted access to MWH use [37]. The majority of MWH users came from the vicinities closer to the location of MWHs, implying that distance is a potential barrier to MWH use for women who reside in remote settings [31] and that women who had to travel for more than 60 minutes were less likely to use MWH [43]. While the use of MWH is promoted as a way to overcome distance and transportation barriers to access skilled birth attendance, women's ability to use MWHs has primarily depended on transportation [16, 31, 33, 37]. The lack of readily accessible and timely transportation to take a women from her residence to MWH and from MWH to hospital considerabily limited the use of existing MWH facilities [16, 31, 33, 37]. The lack of available transportation for referal was a problem that was not alleviated by the exisance of the MWH itself in circumstances when MWHs are not located to the hospital but rather a considerable distance away [13, 31, 43].

#### Financial costs (higher out-of-pocket payments)

Although the Ethiopian government provides free maternity care in public facilities across the country, including stays at MWHs, MWH users face higher out-of-pocket payments than MWH nonusers [40]. The costs include transportation to and from the MWH, meal service during their stay (not all MWHs provide meals to the users), and fees associated with non-medical services [15, 29, 35, 40].

#### The physical aspects of MWHs and services provided

MWHs vary in physical structure, level, and service type. In some areas, MWHs resemble a 'tukul' according to the local and cultural living style [47], and in other parts, they look like a modern corrugated iron house [46]. According to studies, issues relating to the physical structure have been observed, with a single room in particular circumstances required to accommodate more than two women at a time, resulting in users being unable to maintain their privacy [36]. In terms of the services offered by MWHs, some studies have indicated that MWH is a simple structure that provides full accommodation for users, including food catering, water, a place to sleep, ANC care, and health education for pregnant mothers [15, 29]. Whereas other reported that physically, MWHs are available in some areas but do not provide all-encompassing services. As a result, women who planned to stay there were required to bring their food and basic household supplies from home, such as mattresses and kitchen utensils for the duration of their stay [14, 21, 31, 35, 36]. 

#### Cultural restriction

The refusal of a husband or parent to use MWH was demonstrated to be a barrier to MWH use. Pregnant women who desire to stay at MWH are expected to leave their children at home, and there is a societal view that leaving children alone at home without someone to support is a sign of a refusal of social responsibility [21, 31, 35, 36, 40].

#### Poor awareness and women’s perceptions of the quality of care at MWHs

The current review also showed that a lack of knowledge about MWHs and their related benefits was a barrier to staying at MWH [16, 21, 23–26, 28, 31, 35, 36, 39, 40]. Moreover, previous experience has been also cited as a barrier, particularly the poor interaction between health professionals and women at MWH [16, 31].

### Enablers

#### Availability of MWHs

The contribution of MWHs as a crucial link for other maternal health services, as well as their availability as a way to overcome the geographic gap in access to healthcare facilities, were identified as typical enablers in the literature. Women staying at MWH have the opportunity to share experiences with other pregnant mothers, postpartum health education about family planning, infant feeding, and connecting women to the health facility and PNC services for both mothers and newborns [34].

#### Provision of quality and compassionate care

Providing high-quality, compassionate care to mothers at MWH enabled the mother to return to the facility and serve as a role model to other clients [34, 40].

## Discussion

This study synthesized research evidence on MWH implementation, aiming to identify the benefits, barriers and enablers for MWH implementation in Ethiopia. Despite the limited and variable evidence, the implementation of the MWH strategy is an appropriate strategy to improve access to skilled birth attendance for women who reside in rural and remote settings.

According to the included studies, MWH has been shown to have benefits such as improved access to skilled birth attendants, a lower risk of perinatal death, the potential to reduce stillbirth rate, a lower incidence of obstructed labor, and uterine rupture, improved access to maternal health care  [13–15, 22, 47]. Furthermore, it allows healthcare providers to convey health-related information, such as newborn feeding, family planning, and immunization to pregnant women at MWH. It also gives pregnant women the chance to discuss their experiences with one another and adopt behaviors that are crucial for safe motherhood  [21, 31, 36].

Our review found a disparity in the proportion of maternal deaths among MWH users compared with non-users, which could suggest the positive association of making use of MWHs on the reduction of maternal deaths. This finding is consistent with the findings of other systematic reviews and meta-analyses conducted in developing countries  [50, 51]. The result can be explained by the fact that pregnant women who used MWHs had timely access to emergency obstetric care compared to those women who attempted home delivery (without SBA) or were admitted directly to HIs. This could indicate that non-users might have been delayed in accessing emergency obstetric care as a result of geographical barriers like long travel distances to the HIs and lack of transportation  [52]. However, the increased risks of maternal death among non-users might not necessarily be attributable to the non-use of MWH as the studies were not adjusted for confounders. Pregnant women who didn’t stay at MWH may also be disadvantaged in other ways, such as a lack of money or other resources, familial prohibition, or a lack of understanding or education about the need for obstetric care, all of which may be linked to poor maternal health outcomes.

Our review has shown that MWH users had significantly lower rates of uterine rupture than non-users. A possible explanation might be that non-users developed obstetric complications (obstructed labor) due to their late arrival at HIs, leading to rupture of the uterus and subsequent maternal death [13, 14, 47]. Moreover, this finding strengthens the prime concept behind the establishment of MWHs: MWHs help keep pregnant women near HIs with emergency obstetric care; therefore, they are less likely to experience any delay in obtaining emergency care when need be. On the contrary, women who give birth at home or get directly admitted to HIs are more likely to develop complications due to lack of skilled care or delay in reaching the HIs [17]. This is evident in the fact that major childbirth complications, which adversely affect maternal survival, have been reduced in areas where MWH services have been considered as part of maternity care [53].

However, the level of obstetric care available, as well as the quality of care offered at the health facility to which the MWH is linked, all have a role in the improved pregnancy outcome [54, 55]. The poor pregnancy outcomes among pregnant women admitted to the hospital may be also due to substandard care provided at the care facilities after the women arrived  [18, 56]. We further believe that the difference cannot be attributed to the use of MWHs since the confounding variables such as social support, wealth, awareness of MWH services, awareness of the need of delivering at a facility, availability of transportation, and distances to MWHs were not properly controlled in the included studies. These characteristics may have influenced the women’s decision to stay in MWHs, and those who did not may not have had comparable risk to maternal mortality and morbidity outcomes.

For pregnant women living in remote areas where access to maternity services is limited, MWH is widely recommended as a strategy to improve maternal health, but previous studies have shown that the level of utilization of the existing MWHs is lower than expected  [39, 42]. As a result, pregnant women have continued to give birth without the assistance of skilled birth attendants [5]. To improve the current uptake of existing MWH facilities, issues that may negatively affect women’s ability to use MWHs need to be addressed; in particular, greater awareness should be created about the facilities and attention should also be paid to their physical condition and the quality of care they provide [57]. Other studies have also suggested that some interventions like providing the women with food and basic supplies for the duration of their stay while ensuring basic sanitation would significantly improve the uptake of existing MWH services [34]. Besides, fostering a sense of communal ownership, improving women’s education, and promoting the importance of MWHs among health care providers and community leaders may have a significant impact on the sustainability of the service.

Most importantly, the success of the MWH strategy lies in the assumption that women staying in these homes can be transferred to obstetric facilities as soon they start showing signs of labor, where they can get skilled professional care including cesarean delivery at the right time [58]. Therefore, there is a need for functional and standardized referral systems which connect MWHs and obstetric facilities with operative delivery [12].

### Gaps in the literature

Despite the growing popularity of MWHs since the 1970s as an intervention to reduce maternal mortality, no attempt was made to test its effectiveness using strong designs except one conducted in Jimma zone southern Ethiopia [33]. We have conducted this review with currently available evidence to provide important insights and up-to-date information on the national implementation of MWHs strategy with a particular emphasis on the benefits, barriers and enablers of MWH in Ethiopia.

As far as our search is concerned, more than half of the included studies were from southern Ethiopia. Therefore, this review may not be the full representation of the scenario in the context of Ethiopia. The existence of heterogeneity between studies all the observed differences in the outcome variables between the MWH users and non-users cannot be attributed to the use of MWHs. Most of the original studies included in the review did not provide details about how outcome variables were ascertained, wherefore it could be difficult to ascertain that the difference in perinatal and maternal complication and death maternal death between users and non-users were due to MWH use or not use. In addition, it was not also considered the level of care at the care facilities attached to MWHs, the risk status of pregnant women separately as these were likely to impact pregnancy outcomes.

## Conclusion

This study synthesized research evidence on MWH implementation, aiming to identify the benefits, factors and enablers for MWH implementation in Ethiopia. Despite the limited and variable evidence, the implementation of the MWH strategy is an appropriate strategy to improve access to skilled birth attendance for rural and remote women.

Because there are so many different MWH models and so many variations in the baseline characteristics of the population under study, more studies are needed to determine the contribution while accounting for these differences. As research to date has primarily used non-experimental observational study designs, experimental studies, such as randomized controlled trials or quasi-experimental studies, may be best suited for this goal. In addition, studies must look at differences in baseline obstetric risk and intention to give birth in a facility. Demographic characteristics (confounding factors) that could indicate that women who have access to MWHs are better off in many ways, such as socioeconomic status, education, age, and distance from the facility, and therefore it is these factors that are influencing better outcomes.

## Supplementary Information

**Additional file 1.**

**Additional file 2.**

## Data Availability

All data generated during this study are included in this published article and its supplementary information files.

## Abbreviations

MWH: Maternity waiting home
HIs: Health institutions
SSA: sub-Saharan Africa
WHO: World Health Organization
