# Supplementary material for: Benefits, barriers and enablers of maternity waiting homes utilization in Ethiopia: an integrative review of national implementation experience to date
Source: BMC Pregnancy Childbirth. 2022 Sep 2;22:675. doi: 10.1186/s12884-022-04954-y (PMC9438264; doi:10.1186/s12884-022-04954-y)
Supplement: Supplementary file 1 — Additional file 1. [file 12884_2022_4954_MOESM1_ESM.docx]

**Supplementary file 1**

**Search Strategy for PubMed**

We searched titles and abstracts, adapting the following terms to meet each database’s search requirements: Maternity waiting homes utilization in Ethiopia: an integrative review of national implementation experience to date

| Database | Query | Search # | Search Details | Results |
| --- | --- | --- | --- | --- |
| Pub-Med | #4 AND #5 | 6 | ("Health Services Accessibility"[MeSH Terms] OR "maternity waiting home*"[Text Word] OR "maternity waiting area*"[Text Word] OR "maternity waiting shelter*"[Text Word] OR "antenatal villag*"[Text Word] OR "maternity waiting facilit*"[Text Word] OR "maternity waiting*"[Text Word]) AND ("maternal mortality"[MeSH Terms] OR "maternal mortality"[Text Word] OR "maternal Health"[MeSH Terms] OR "maternal Health"[Text Word] OR "perinatal mortality"[MeSH Terms] OR "perinatal mortality"[Text Word] OR "pregnancy outcome"[MeSH Terms] OR "maternal outcome*"[Text Word] OR "perinatal outcome*"[Text Word]) AND (("Health Services Accessibility"[MeSH Terms] OR "maternity waiting home*"[Text Word] OR "maternity waiting area*"[Text Word] OR "maternity waiting shelter*"[Text Word] OR "antenatal villag*"[Text Word] OR "maternity waiting facilit*"[Text Word] OR "maternity waiting*"[Text Word]) AND ("Ethiopia"[MeSH Terms] OR "Ethiopia"[Text Word])) | 176 |
|  | #1 AND #3 | 5 | ("Health Services Accessibility"[MeSH Terms] OR "maternity waiting home*"[Text Word] OR "maternity waiting area*"[Text Word] OR "maternity waiting shelter*"[Text Word] OR "antenatal villag*"[Text Word] OR "maternity waiting facilit*"[Text Word] OR "maternity waiting*"[Text Word]) AND ("Ethiopia"[MeSH Terms] OR "Ethiopia"[Text Word]) | 562 |
|  | #1 AND #2 | 4 | ("Health Services Accessibility"[MeSH Terms] OR "maternity waiting home*"[Text Word] OR "maternity waiting area*"[Text Word] OR "maternity waiting shelter*"[Text Word] OR "antenatal villag*"[Text Word] OR "maternity waiting facilit*"[Text Word] OR "maternity waiting*"[Text Word]) AND ("maternal mortality"[MeSH Terms] OR "maternal mortality"[Text Word] OR "maternal Health"[MeSH Terms] OR "maternal Health"[Text Word] OR "perinatal mortality"[MeSH Terms] OR "perinatal mortality"[Text Word] OR "pregnancy outcome"[MeSH Terms] OR "maternal outcome*"[Text Word] OR "perinatal outcome*"[Text Word]) | 3,412 |
|  | "Ethiopia"[MeSH Terms] OR "Ethiopia"[Text Word] | 3 | "Ethiopia"[MeSH Terms] OR "Ethiopia"[Text Word] | 23,262 |
|  | "maternal mortality"[MeSH Terms] OR "maternal mortality"[Text Word] OR "maternal Health"[MeSH Terms] OR "maternal Health"[Text Word] OR "perinatal mortality"[MeSH Terms] OR "perinatal mortality"[Text Word] OR "pregnancy outcome"[MeSH Terms] OR "maternal outcome*"[Text Word] OR "perinatal outcome*"[Text Word] | 2 | "maternal mortality"[MeSH Terms] OR "maternal mortality"[Text Word] OR "maternal Health"[MeSH Terms] OR "maternal Health"[Text Word] OR "perinatal mortality"[MeSH Terms] OR "perinatal mortality"[Text Word] OR "pregnancy outcome"[MeSH Terms] OR "maternal outcome*"[Text Word] OR "perinatal outcome*"[Text Word] | 126,157 |
|  | (("Health Services Accessibility"[MeSH Terms] OR "maternity waiting home*"[Text Word] OR "maternity waiting area*"[Text Word] OR "maternity waiting shelter*"[Text Word] OR "antenatal villag*"[Text Word] OR "maternity waiting facilit*"[Text Word] OR "maternity waiting*"[Text Word])) | 1 | "Health Services Accessibility"[MeSH Terms] OR "maternity waiting home*"[Text Word] OR "maternity waiting area*"[Text Word] OR "maternity waiting shelter*"[Text Word] OR "antenatal villag*"[Text Word] OR "maternity waiting facilit*"[Text Word] OR "maternity waiting*"[Text Word] | 120,044 |
